# Spatiotemporal analysis of air pollution and asthma patient visits in Taipei, Taiwan

**DOI:** 10.1186/1476-072X-8-26

**Published:** 2009-05-07

**Authors:** Ta-Chien Chan, Mei-Lien Chen, I-Feng Lin, Cheng-Hua Lee, Po-Huang Chiang, Da-Wei Wang, Jen-Hsiang Chuang

**Affiliations:** 1 National Health Command Center, Centers for Disease Control, Taipei, Taiwan, R.O.C; 2 Institute of Epidemiology, College of Public Health, National Taiwan University, Taipei, Taiwan, R.O.C; 3 Institute of Environmental and Occupational Health Sciences & Department of Environmental and Occupational Medicine, School of Medicine, National Yang-Ming University, Taipei, Taiwan, R.O.C; 4 Department of Social Medicine, School of Medicine, National Yang-Ming University, Taipei, Taiwan, R.O.C; 5 Institute of Public Health, School of Medicine, National Yang-Ming University, Taipei, Taiwan, R.O.C; 6 Institute of Health Care Administration, National Yang-Ming University, Taipei, Taiwan, R.O.C; 7 Center for Health Policy Research and Development, National Health Research Institutes, Miaoli County, Taiwan, R.O.C; 8 Bureau of National Health Insurance, Taipei, Taiwan, R.O.C; 9 Institute of BioMedical Informatics, School of Medicine, National Yang-Ming University, Taipei, Taiwan, R.O.C; 10 Institute of Information Science, Academia Sinica, Taipei, Taiwan, R.O.C

## Abstract

**Background:**

Buffer analyses have shown that air pollution is associated with an increased incidence of asthma, but little is known about how air pollutants affect health outside a defined buffer. The aim of this study was to better understand how air pollutants affect asthma patient visits in a metropolitan area. The study used an integrated spatial and temporal approach that included the Kriging method and the Generalized Additive Model (GAM).

**Results:**

We analyzed daily outpatient and emergency visit data from the Taiwan Bureau of National Health Insurance and air pollution data from the Taiwan Environmental Protection Administration during 2000–2002. In general, children (aged 0–15 years) had the highest number of total asthma visits. Seasonal changes of PM_10_, NO_2_, O_3 _and SO_2 _were evident. However, SO_2 _showed a positive correlation with the dew point (r = 0.17, p < 0.01) and temperature (r = 0.22, p < 0.01). Among the four pollutants studied, the elevation of NO_2 _concentration had the highest impact on asthma outpatient visits on the day that a 10% increase of concentration caused the asthma outpatient visit rate to increase by 0.30% (95% CI: 0.16%~0.45%) in the four pollutant model. For emergency visits, the elevation of PM_10 _concentration, which occurred two days before the visits, had the most significant influence on this type of patient visit with an increase of 0.14% (95% CI: 0.01%~0.28%) in the four pollutants model. The impact on the emergency visit rate was non-significant two days following exposure to the other three air pollutants.

**Conclusion:**

This preliminary study demonstrates the feasibility of an integrated spatial and temporal approach to assess the impact of air pollution on asthma patient visits. The results of this study provide a better understanding of the correlation of air pollution with asthma patient visits and demonstrate that NO_2 _and PM_10 _might have a positive impact on outpatient and emergency settings respectively. Future research is required to validate robust spatiotemporal patterns and trends.

## Background

Asthma remains a major health issue for children in Taiwan [1,2]. Taipei City is a highly urbanized area with crowded population density (9,720 people/km^2^) [3] and intensive motorcycle and sedan density (motorcycles: 3,927 vehicles/km^2^; sedan: 2,672 vehicles/km^2^) [4]. Due to this heavy traffic condition, the estimated child asthma prevalence in Taipei City is 13% and the trend is becoming increasingly more serious [5]. Known risk factors for asthma include many external determinants such as mites, dust, air pollution, weather conditions and so on [1,2,6,7]. Associations between short term exposure to ambient air pollutants and health outcomes have also been reported, based on limited spatial and temporal information on pollution sources and concentration [5,8-11]. Within this research, exposure assessment may be the most critical analytic tool.

Recently, geographic information system (GIS) has been applied to estimate the concentration of air pollutants [12] and many epidemiologic studies have adopted GIS to explore the health impact of air pollutants on asthma [11,13,14]. Buffer analysis with data from air monitoring stations and proximity analysis to ambient pollution sources near the highways or busy roads are also frequently used. However, little is known about how air pollutants affect health outside a defined buffer. Therefore, we hypothesized that the localized level of air pollution concentration might have different effects on asthma visits. Although different districts in Taipei City might have different concentrations, it was not feasible to set up the air monitoring stations in each district. In order to make an exposure assessment for the whole of Taipei City, we linked the daily exposure level by geostatistical method and corresponding asthma visits to estimate the impact on asthma visits by air pollutants.

We investigated the association between air pollution and asthma patient visits in Taipei, Taiwan, with two main objectives. First, we estimated the pollutant level by constructing a spatial and temporal model representing a geographical area using daily average pollutant concentration data. Second, we linked air pollutant concentration to asthma outpatient and emergency visits within the defined metropolitan area. We hypothesized that there would be a direct relationship between the amount of air pollution and the number of asthma patient visits.

## Results

Taipei City, with very high population density, had approximately 2.64 million residents during 2000–2002. The sex ratio (male/female) was 0.97/1.00 and the age distribution was 0–15 years (20%), 16–65 years (70.4%), and > 65 years (9.6%). The total area of Taipei City is 271.8 (km^2^). Demographic information for each district in 2000 is listed in Table 1[3]. During 2000–2002, asthma patient visits included a total of 724,075 outpatient visits and 34,274 emergency visits. A slightly higher percentage of male visits were observed for the emergency visits (58.5%) than outpatient visits (55.8%). In these two settings, children (0–15 years) had the highest number of total asthma visits (outpatient: 48.8%, emergency: 46.1%) and those older than 65 years had the lowest number of total visits (outpatient: 16.8%; emergency: 15.1%).

**Table 1:** Demographic data of Taipei City in 2002

District Name	Age 0–15	Age 16–65	Age >= 66	All-Age	Area (km^2^)	Population Density (persons/km^2^)
Beitou District	50,358	177,023	21,734	249,115	56.82	4,384
Da-an District	62,101	217,859	35,754	315,714	11.36	27,788
Datong District	24,645	93,272	13,160	131,077	5.68	23,071
Jhongjheng District	34,263	108,935	18,610	161,808	7.61	21,271
Jhongshan District	39,506	156,737	21,326	217,569	13.68	15,902
Nangang District	23,583	81,138	9,118	113,839	21.84	5,212
Neihu District	60,192	181,567	16,852	258,611	31.58	8,189
Shihlin District	56,691	209,152	25,650	291,493	62.37	4,674
Sinyi District	44,197	168,018	25,147	237,362	11.21	21,178
Songshan District	42,959	142,378	19,952	205,289	9.29	22,103
Wanhua District	35,648	144,357	23,446	203,451	8.85	22,983
Wunshan District	54,712	178,647	23,169	256,528	31.51	8,141

Total	528,855	1,859,083	253,918	2,641,856	271.8	9,720

Sources: Taipei City Government

**Figure 1:**
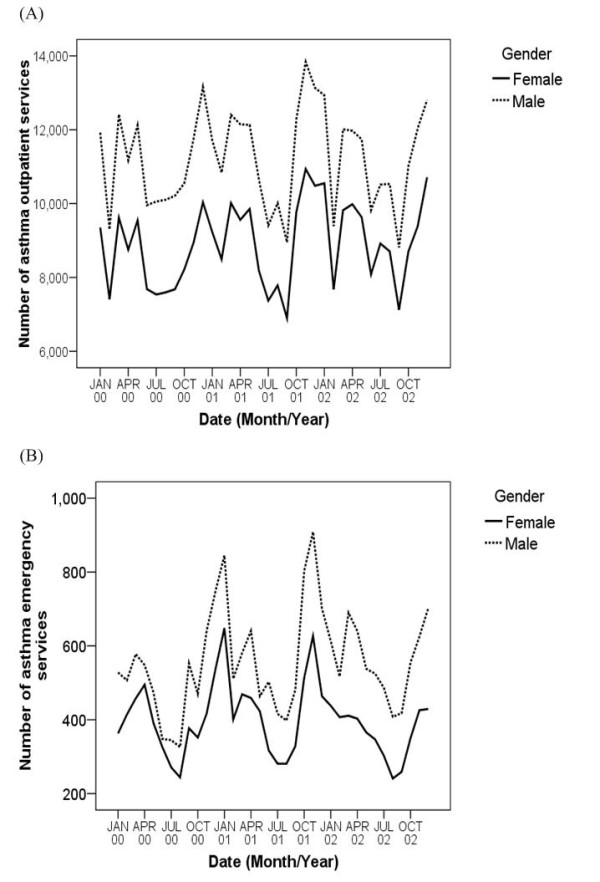
**Monthly asthma (A) outpatient services and (B) emergency services according to gender groups**.

The data indicated that March and December were the two significant peak periods (Figure 1) for both asthma outpatient and emergency visits. Gender-specific monthly asthma outpatient and emergency visits are shown in Figure 1, illustrating the similarity in seasonal variation for both genders. Males consistently had a higher number of visits than females.

**Figure 2:**
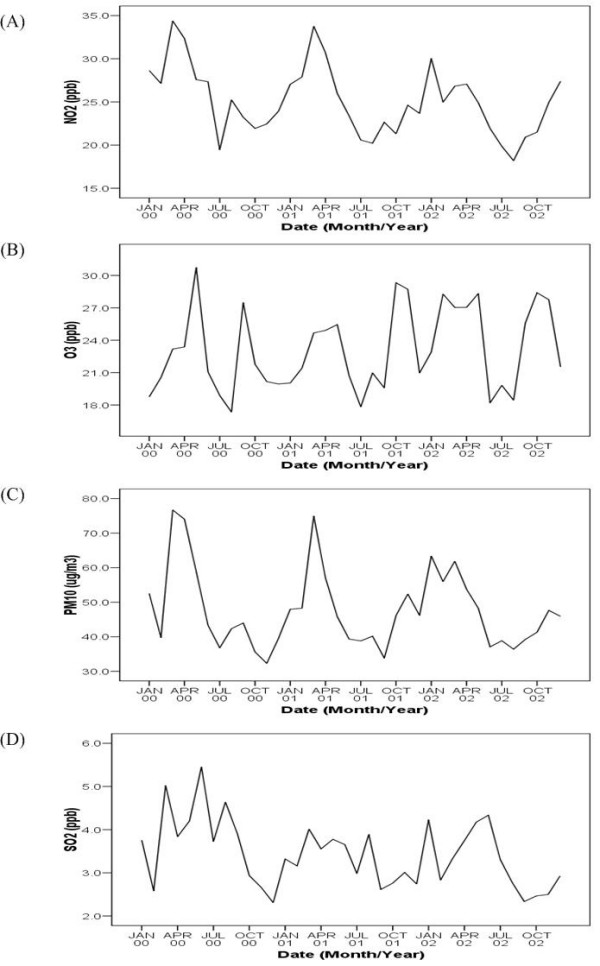
**Mean concentration trends of four air pollutants**.

Overall, PM_10_, NO_2_, O_3_, SO_2 _showed significant seasonal changes (Figure 2). The monthly NO_2 _average concentration had only one wave of increase around March each year. PM_10 _and O_3 _had the largest seasonal variation with two-fold concentration increases in certain months during the same period. SO_2 _had a different seasonal pattern than other pollutants. It had a higher concentration in the summer rather in the spring. The correlation among these four pollutants is shown in Table 2. The highest positive correlation was between NO_2 _and SO_2 _(r = 0.63, p < 0.01). Weather conditions, including dew point and ambient temperature, had negative correlations among PM_10_, NO_2 _and O_3_. We found positive correlations on all age asthma outpatient visits among PM_10_, NO_2 _and SO_2_. Dew point and temperature had negative correlations on asthma visits. The spatial distribution estimated by the Kriging method showed a high concentration of air pollution in downtown Taipei City (Table 3). The largest variation in spatial concentration among the four pollutants was NO_2_, possibly caused by a high volume of traffic.

**Table 2:** Correlation between asthma outpatient visits and air pollutants, weather conditions.

Asthma Visits	PM_10_	SO_2_	O_3_	NO_2_	Dew point	Temperature
Asthma Visits 1	0.13**	0.15**	-0.02	0.25**	-0.16**	-0.17**
PM_10_	1	0.55**	0.31**	0.59**	-0.25**	-0.15**
SO_2_		1	0.01	0.63**	0.17**	0.22**
O_3_			1	-0.02	-0.26**	-0.1**
NO_2_				1	-0.16**	-0.22**
Dew point					1	0.91**
Temperature						1

** Correlation is significant at the 0.01 level (2-tailed).

Asthma Visits: All age asthma outpatient visits.

**Table 3:** 3-Year (2000~2002) Average of Air Pollution Concentration in each district

District Name	PM_10 _(95% CI)	SO_2 _(95% CI)	O_3 _(95% CI)	NO_2 _(95% CI)
Beitou	42.53 (41.18~44.97)	1.59 (1.49~1.68)	50.36 (49.17~53.27)	14.3 (13.72~15.11)
Da-an*	45.10 (43.63~47.68)	3.26 (3.15~3.45)	50.42 (48.77~53.31)	28.47 (27.83~30.12)
Datong*	54.49 (52.73~57.61)	3.43 (3.31~3.63)	46.91 (45.49~49.6)	29.15 (28.6~30.85)
Jhongjheng*	46.44 (44.94~49.1)	2.92 (2.81~3.08)	49.76 (48.15~52.61)	27.69 (27.21~29.3)
Jhongshan*	52.37 (50.67~55.37)	3.13 (3.01~3.31)	47.38 (45.95~50.1)	28.41 (27.89~30.06)
Nangang	46.56 (45.13~49.23)	3.61 (3.48~3.82)	50.06 (48.53~52.93)	26.23 (25.58~27.75)
Neihu	46.79 (45.36~49.48)	2.61 (2.47~2.76)	49.09 (47.65~51.91)	22.91 (22.1~24.21)
Shihlin	44.58 (43.19~47.14)	1.88 (1.76~1.98)	50.19 (48.89~53.08)	17.27 (16.57~18.25)
Sinyi	45.96 (44.52~48.6)	3.8 (3.67~4.02)	50.4 (48.76~53.29)	29.09 (28.31~30.76)
Songshan*	50.32 (48.73~53.21)	3.33 (3.2~3.52)	48.36 (46.84~51.14)	28.9 (28.36~30.58)
Wanhua*	45.32 (43.83~47.91)	2.97 (2.85~3.14)	50.52 (48.9~53.41)	25.84 (25.3~27.34)
Wunshan	41.89 (40.59~44.29)	2.96 (2.85~3.13)	53.12 (51.47~56.17)	22.88 (22.17~24.19)

*Downtown Area in Taipei City

Tables 4, 5 represent the different patterns of the effects of a 10% increase in pollutant concentration on outpatient and emergency visits respectively, as estimated by the Generalized Additive Model (GAM). In outpatient visits, as the lag days increased, the air pollution's effects on asthma outpatient visits decreased except for O_3 _by model 1(single pollutant model). In model 2 (four pollutants model), after adjusting for the other pollutants, the average effects of the pollutants on outpatient visits were all decreased. At 0-day lag, the highest effects on outpatient visits were NO_2 _and SO_2 _in model 2, which was consistent with the results in model 1. In model 2 (Table 4), at 0-day lag, the mean effect of a 10% increase in NO_2 _on the change of outpatient visits was 0.3 (95% CI: 0.16%~0.45%) and the effects ranged from -0.06% to 0.94% among the 12 districts. But the pattern was reversed for emergency visits where the effect was not observed until after a 1-day lag. In emergency visits, PM_10 _and SO_2 _had a positive effect with statistical significance on asthma emergency visits at the 2-day lag by model 1. In model 2, only PM_10 _had a positive effect with statistical significance on asthma emergency visits at the 2-day lag. In model 2 (Table 5), at 2-day lag, the mean effect of a 10% increase in PM_10 _on the change of emergency visits was 0.53 (95% CI: 0.27%~0.79%) and the effects ranged from -0.37% to 1.20% among the 12 districts.

**Table 4:** Effect of 10% increase in pollutant concentration on asthma outpatient visits (%)

	0-day lag			1-day lag			2-day lags		
		
Mode 1									
Pollutants	Mean	95% LCI	95% UCI	Mean	95% LCI	95%UCI	Mean	95% LCI	95%UCI
PM_10_	0.34*	0.22	0.46	0.14*	0.02	0.26	0.19*	0.11	0.27
SO_2_	0.44*	0.31	0.57	0.25*	0.16	0.35	0.13*	0.02	0.24
O_3_	0.08*	0.03	0.14	0.13*	0.09	0.18	0.11*	0.05	0.16
NO_2_	0.65*	0.48	0.83	0.24*	0.06	0.42	0.11	-0.08	0.29

Model 2									

PM_10_	0.20*	0.01	0.39	-0.05	-0.18	0.09	0.14*	0.01	0.28
SO_2_	0.27*	0.12	0.41	0.19*	0.05	0.32	0.03	-0.12	0.18
O_3_	-0.13*	-0.24	-0.01	0.06	-0.01	0.12	0.07*	0.00	0.15
NO_2_	0.30*	0.16	0.45	-0.03	-0.30	0.25	0.00	-0.25	0.24

◎ Model 1: Single pollutant model.

Model 2: Four pollutants model.

*Statistically significant.

**Table 5:** Effect of 10% increase in pollutant concentration on asthma emergency visits (%)

	0-day lag			1-day lag			2-day lags		
		
Model 1									
Pollutants	Mean	95% LCI	95% UCI	Mean	95% LCI	95%UCI	Mean	95% LCI	95%UCI
PM_10_	0.03	-0.24	0.29	0.15	-0.16	0.46	0.43*	0.22	0.65
SO_2_	-0.06	-0.27	0.14	-0.01	-0.20	0.19	0.17*	0.02	0.33
O_3_	-0.06	-0.24	0.13	0.00	-0.09	0.09	0.05	-0.07	0.17
NO_2_	-0.09	-0.47	0.28	0.16	-0.10	0.42	-0.05	-0.37	0.27

Model 2									

PM_10_	0.14	-0.17	0.44	0.16	-0.21	0.52	0.53*	0.27	0.79
SO_2_	-0.02	-0.26	0.21	-0.06	-0.28	0.17	0.10	-0.14	0.34
O_3_	-0.08	-0.25	0.09	-0.12	-0.19	-0.05	-0.04	-0.25	0.16
NO_2_	-0.15	-0.43	0.13	0.10	-0.19	0.38	-0.43	-0.86	0.00

◎ Model 1: Single pollutant model.

Model 2: Four pollutants model.

*Statistically significant.

In general, those districts in Taipei City with a higher concentration of air pollutants had a significant increase in asthma outpatient visits. At 0-day lag, the elevation of NO_2 _concentration had the highest impact on asthma outpatient visits on the day that a 10% increase of its concentration caused the asthma outpatient visit rates to increase by 0.65% (95% CI: 0.48%~0.83%). SO_2_'s effect on outpatient visits was 0.44% (95% CI: 0.31%~0.57%). PM_10_'s effect on outpatient visits was 0.34% (95% CI: 0.22%~0.46%). O_3 _had a minor effect on outpatient visits. At the 1-day lag, the elevation of 4 pollutants' concentration all had a significant increase on outpatient visits. After adjusting for the other 3 pollutants, SO_2 _still had an effect on outpatient visits. At the 2-day lag, the elevation of PM_10 _and O_3_'s concentration had a significant increase on outpatient visits after adjusting for the other pollutants (Table 4). Table 5 demonstrates how the pattern of air pollutants' effect on emergency visits was different from the effect on outpatient visits. At the 0-day lag, all four air pollutants showed non-significant effects on emergency visits. Until the 2-day lag, PM_10 _had the most significant influence on emergency visits with an increase by 0.53% after adjusting for the other pollutants (95% CI: 0.27%~0.79%).

We examined air pollution's impacts on 3 age-related groups: children (0 – 15 years), adults (16 – 65 years), and elderly (> 65 years). Table 6 demonstrates the overall effects on outpatient and emergency visits in Taipei City at the 0-day lag. Within outpatient visits, children were more sensitive to the elevation of NO_2 _and PM_10_. In general, NO_2 _had the highest effect on outpatient visits, even after adjusting for the other pollutants. Within emergency visits, children were still more sensitive to the elevation of NO_2 _and PM_10_, but the effect was not statistically significant.

**Table 6:** Age-specific effect of 10% increase in pollutant concentration on asthma outpatient and emergency visits (%) in Taipei City at 0-day lag

	Outpatient (0-day lag)			
	
Model-1	PM_10 _(95% CI)	SO_2 _(95% CI)	O_3 _(95% CI)	NO_2 _(95% CI)
Age 0–15	0.41 (0.13~0.69)*	0.28 (0.08~0.47)*	0.08 (-0.05~0.22)	0.66 (0.21~1.11)*
Age 16–65	0.43 (0.30~0.55)*	0.51 (0.36~0.66)*	0.22 (0.11~0.33)*	0.88 (0.65~1.11)*
Age >= 66	0.15 (0.00~0.29)*	0.36 (0.23~0.49)*	0.06 (-0.05~0.18)	0.29 (0.08~0.49)*
All Age	0.34 (0.22~0.46)*	0.44 (0.31~0.57)*	0.08 (0.03~0.14)*	0.65 (0.48~0.83)*

	Outpatient (0-day lag)			
	
Model-2	PM_10 _(95% CI)	SO_2 _(95% CI)	O_3 _(95% CI)	NO_2 _(95% CI)

Age 0–15	0.26 (-0.10~0.63)	0.12 (-0.20~0.44)	-0.15 (-0.24~-0.05)*	0.22 (-0.15~0.59)
Age 16–65	0.24 (0.08~0.39)*	0.20 (0.04~0.36)*	-0.07 (-0.26~0.11)	0.46 (0.27~0.65)*
Age >= 66	0.07 (-0.10~0.25)	0.33 (0.19~0.47)*	-0.03 (-0.19~0.14)	0.03 (-0.25~0.32)
All Age	0.20 (0.01~0.39)*	0.27 (0.12~0.41)*	-0.13 (-0.24~-0.01)*	0.30 (0.16~0.45)*

	Emergency (0-day lag)			
	
Model-1	PM_10 _(95% CI)	SO_2 _(95% CI)	O_3 _(95% CI)	NO_2 _(95% CI)

Age 0–15	0.12 (-0.44~0.67)	-0.04 (-0.24~0.16)	0.09 (-0.19~0.38)	0.17 (-0.30~0.64)
Age 16–65	-0.17 (-0.55~0.22)	-0.09 (-0.36~0.17)	-0.08 (-0.24~0.09)	-0.21 (-0.60~0.18)
Age >= 66	0.23 (-0.07~0.53)	-0.26 (-0.50~-0.01)*	-0.01 (-0.15~0.13)	-0.03 (-0.21~0.15)
All Age	0.03 (-0.24~0.29)	-0.06 (-0.27~0.14)	-0.06 (-0.24~0.13)	-0.09 (-0.47~0.28)

	Emergency (0-day lag)			
	
Model-2	PM_10 _(95% CI)	SO_2 _(95% CI)	O_3 _(95% CI)	NO_2 _(95% CI)

Age 0–15	-0.04 (-0.42~0.34)	-0.01 (-0.35~0.33)	0.05 (-0.21~0.30)	-0.08 (-0.58~0.41)
Age 16–65	-0.08 (-0.53~0.37)	0.03 (-0.22~0.27)	-0.01 (-0.18~0.16)	-0.19 (-0.58~0.20)
Age >= 66	0.25 (-0.13~0.64)	-0.30 (-0.64~0.05)	0.03 (-0.17~0.22)	-0.01 (-0.37~0.35)
All Age	0.14 (-0.17~0.44)	-0.02 (-0.26~0.21)	-0.08 (-0.25~0.09)	-0.15 (-0.43~0.13)

*Model 1: Single pollutant model.

Model 2: Four pollutants model.

*Statistically significant.

## Discussion

### Characteristics of this study

This research used GIS software with the Kriging method to estimate Taipei City's air pollution concentration in Metropolitan Taipei. Although other researchers have employed a similar method to evaluate the concentration of pollutants, they did not use such approaches to calculate the daily concentration and exposure to air pollutants in different districts, as was the case in this study. In addition, this study used the GAM to examine the relationship between air pollutants and asthma. The combined use of the above methods allowed us to improve on past studies [13,15], which focused on smaller and more limited areas. The integrated methods we used allowed an assessment of the health effects of air pollution in a wider area, which might be useful for exposure assessments of air pollution.

### Age and gender distribution of outpatient and emergency visits

Our study illustrated a significant seasonal variation within outpatient and emergency visits, especially in the spring and winter. Males, who accounted for 55.8% of outpatient visits and 58.5% of emergency visits, and young children, who accounted for 48.8% of outpatient visits and 46.1% of emergency visits, had a higher incidence of medical visits related to asthma. These findings are consistent with other asthma studies in Taiwan [15,16].

### Different patterns of outpatient and emergency visits affected by air pollutants

In outpatient settings, the main effect of air pollutants occurred on the first two days of exposure. When we compared model 1 with model 2, the adjusted effects had slightly declined due to the same direction of the effects. Downtown Taipei City, more than any other areas in Metropolitan Taipei, had a higher rate of increase for asthma emergency visits for the same time period. For Taipei City as a whole, when the concentration of air pollutants increased by 10%, there appeared to be an initial decrease in emergency visits, followed by an increase at the 2-day lag, suggesting a lag effect of air pollution on patient visits to hospital emergency departments. The possible explanation for this phenomenon is that because asthma is a chronic illness, patients were experienced in dealing with their symptoms. When air pollutant concentration was elevated, patients with asthma may have self-treated their symptoms or gone to neighbourhood clinics and hospital outpatient departments for medical treatment. Subsequently, if patients did not have any treatment or if the outpatient visit was ineffective, they would then go to hospital emergency departments for assistance. This would explain why the increase in emergency visits was delayed.

### Comparison with other studies

We compared our findings with two other studies [17-19]. Hwang and Chan, focusing on patients with lower respiratory tract diseases, used a 2-stage spatio-temporal model. The second stage of this model, also used by Dominici et al[17], estimated whether air pollutant concentration had any influence on patients with lower respiratory tract disease, and which resulted in them seeking medical treatment. Hwang and Chan's cases were selected from air quality monitoring stations and all the community clinics surrounding these stations. Sampling points included 50 townships across Taiwan. Hwang and Chan's findings concerning the percentage change in outpatient visits paralleled the findings of our study in Taipei City.

When they evaluated the impact of air pollutants, Hwang and Chan reported that NO_2 _was the pollutant that influenced the most number of patient visits by people with respiratory tract diseases and they noted that SO_2_, O_3 _and PM_10 _all had an impact on outpatient visits. We also found that all four air pollutants had a positive effect on asthma outpatient visits in model 1. The PM_10 _had significant impact on asthma emergency visits after 2 days' exposure.

Dominici et al. [20] observed an increase in hospitalization for cardiovascular and respiratory tract diseases, noting that rates increased with increments of every 10 μg/m^3 ^in PM_2.5_. The two pulmonary diseases studied by Dominici et al. were chronic obstructive pulmonary disease (COPD) and respiratory tract infection. For COPD, the hospital visit rate increased 0.91% at the 0-day and 1-day lags; but at the 2-day lag, the rate decreased to 0.3%, and it was not significant in the statistics. There are similarities in asthma outpatient visits between Dominici et al.'s finding and our study. For respiratory tract infection, Dominici et al. reported that the effect of PM_2.5 _was not obvious from the 0-day to 1-day lags, but the rate increased to 0.92% at the 2-day lag, which also parallels our findings in emergency setting.

### Limitations

This study used districts' daily average level of pollutants as the population's exposure level; when the workplace was not located in the same district as the home there could be bias about an individual's exposure estimation, which could influence the results. In addition, the districts where outpatient visits and emergency visits took place were assumed to be the same districts where people were exposed to pollution. This may not always have been the case. The true exposure time was difficult to estimate due to lack of exposure information. There might be misclassification of exposure due to the duration between exposure time and hospital/clinic visits' time [21]. Although we have considered the lag effect, the strength might be underestimated at a different lag day. We also considered the reliability of diagnostic codes in the claim data and the medical records. Based on an unpublished study in Taiwan and another study in Canada [22], the reliability of asthma diagnosis was high, but we observed that prevalence was underestimated. In constructing the interpolation model of air pollutants, we were constrained by a limited number of air monitoring stations. In the northern side of Taipei city, there was a mountain area which did not have any air monitoring stations, which might cause prediction error. There were also many environmental factors affecting the distribution of air pollutants, such as wind direction and wind speed, which were not considered in this current study.

## Conclusion

In conclusion, this preliminary study illustrates the potential use of the Kriging method and GAM to evaluate the effects of air pollution on asthma patient visits. The results of this study provide a better understanding of the correlation of air pollution on asthma patient visits and demonstrate that NO_2 _and PM_10 _might have a positive impact each on outpatient and emergency settings respectively. Future research is required to provide robust spatiotemporal patterns and trends.

## Methods

### Patient visit data source and definitions

This study used computerized claims data from the Bureau of National Health Insurance, which provides comprehensive health insurance coverage (99%) of the 23 million people in Taiwan, with service dates from January 2000 to December 2002, totalling 1096 days. In compliance with the Personal Electronic Data Protection Law in Taiwan, no identifiable personal data were used. We selected patient visit data with a diagnosis of asthma (*International Classification of Diseases, Ninth Revision, Clinical Modification code *493.0–493.2 and 493.9). An asthma outpatient visit was defined as a patient visit to a physician's office, clinic, or hospital outpatient department with the diagnosis coded as asthma. An asthma emergency visit was defined as a patient visit to a hospital emergency department with the diagnosis coded as asthma. Each occurrence, limited to Taipei City, was counted as one visit. We excluded potentially miscoded data pertaining to patient visits to clinics or departments, such as dentistry, dermatology, ophthalmology, obstetrics and gynaecology, and traditional Chinese medicine, unlikely to have asthma as a diagnosis. The institutional review board of National Yang-Ming University, Taipei, Taiwan approved the study.

### Data processing

The basic geographic unit for this study was an administrative "district" under the Taipei city government, with 12 districts in total. All data were aggregated by district and compared to the daily concentration of pollutants for each district.

### Air pollution data and spatial mapping

**Figure 3:**
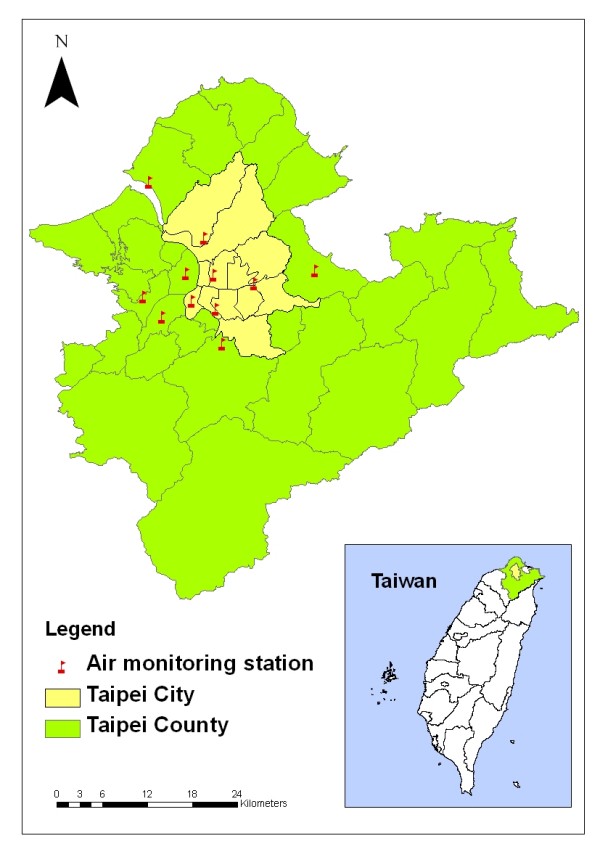
**Air monitoring stations in Taipei City and Taipei County**.

Measurements of air pollutants were based on data routinely collected at 11 Environmental Protection Administration (EPA) monitoring stations: five in Taipei City and six in Taipei County (Figure 3). Each monitoring station provided hourly readings of the concentration of the gaseous pollutants SO_2_, NO_2_, O_3_, and ambient PM with an aerodynamic diameter ≦ 10 *μ*m (PM_10_) together with weather condition related data, such as temperatures and dew points.

With the data from each monitoring station, the Ordinary Kriging method was used to estimate the pollutant levels of each district by date from January 2000 to December 2002 for each of the four pollutants: SO_2_, NO_2_, O_3_, and PM_10_. In general, the Kriging method [12,23] was used as a statistical mapping technique using data collected at each point location, to predict concentration in each grid cell over a spatial domain. We used Spatial Analyst and Geostatistical Analyst extension of ArcGIS (ArcMap, version9.0; ESRI Inc., Redlands, CA, USA) using 0.086 km by 0.086 km grids to partition each district for each pollutant and each day. When modelling Kriging, there were some parameters that had to be selected including partial sill, range, nugget effect and semivariogram [24]. We identified the day of highest concentration in each air pollutant for the model selection. Our assumption was that higher concentrations would affect a broader area, allowing us to determine the maximum range. Nugget effect was a kind of measurement error that we assumed to be zero. We used three kinds of semivariogram including spherical, exponential and Gaussian models to examine the best fit of the data. Partial sill was determined after deciding the above parameters. Average prediction error (PE) and root mean square standardized (RMSS)[12] was used to select which model was the best to estimate the distribution of air pollutants. The parameters of semivariogram used in this study are listed [see Additional file 1]. The cross-validation of the four air pollutants was done manually by ArcGIS Geostatistical extension [see Additional file 2]. The criteria for a good-fitting Kriging model used in this study were an average PE near 0 and RMSS near 1. According to the cross-validation results, if RMSS < 1, there was tendency toward overestimating the variance [25], in the cases of SO_2 _and O_3_; if RMSS > 1, there was tendency toward underestimation [25] in the cases of PM_10 _and NO_2_.

**Figure 4:**
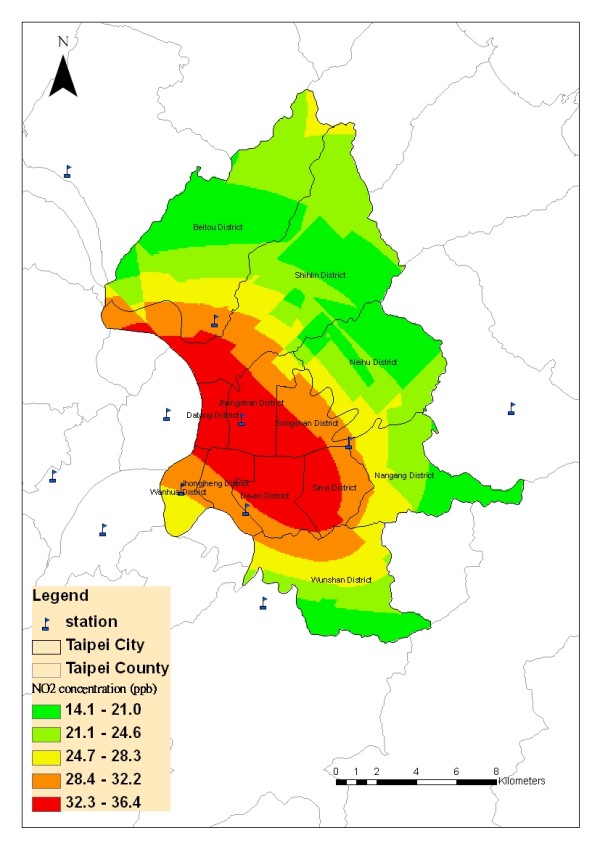
**An example of estimated daily air pollution levels using air monitoring data and Kriging in Taipei City, Taiwan**.

After defining all parameters, Python script and Model Builder were employed to handle batching calculations of daily concentration of pollutants. The automatic outputs of air pollutants' concentration were recoded in 1096 daily ".dbf" files in each pollutants and SAS macro was applied to combine all files. Figure 4 shows an example of spatial distribution of NO_2 _predicted with the Ordinary Kriging derived from the data measured by the 11 monitoring stations which were located in Taipei city and surrounding Taipei County.

### Exposure assessment

Due to privacy issues, the patients' exact addresses were not provided. Therefore, we had to make assumptions about the location of exposures. The district where a patient visit took place was taken as the geographical area where the patient was most likely exposed to air pollutants. Each asthma patient visit was matched with the district's daily 24-hour average pollutant concentration for that date. As patients may not have had any asthma symptoms, resulting in a visit to hospital, clinic or emergency department until one or two days after being exposed to pollutants, the possible time lag was considered. The 10% increase of air pollutant, PM_10 _was shown as an example to express the elevation of concentration in our effect's calculation [see Additional file 3].

### Statistical analysis

The study sought to investigate an association between air pollutants and asthma in two aspects: temporal and spatial exposure. Statistical analysis used the number of patient visits as the dependent variable and average daily concentration of ambient SO_2_, NO_2_, O_3 _and PM_10 _as the independent variables. We also took into consideration the effect of weather conditions, including daily dew point and temperature [19] and the decrease of patient visits attributed to extended holidays, such as Chinese New Year. The analysis used GAM [17,18] a nonparametric smoothing method, to examine the association between the group level dependent variable and independent variables. We assumed a Poisson GAM with log link function and used the cubic smoothing spline method [26] to fit the model. We calculated the parameters of air pollutants in different age groups (0–15, 16–65, > 65, and all ages) after making adjustments to address influences by weekend and weekday effects, weather conditions including dew point and temperature, extended holidays, and population in each district. There were 2 models, including the single pollutant model (model 1) and the four pollutants model (model 2), in the final calculation and 6 confounders in the models (the two models were described in the additional file [see Additional file 4]). The only difference was the number of air pollutants. In model-1, each model only contains one air pollutant. In model-2, four air pollutants were included.

### Health effect

Considering that air pollutants may have a lag effect on asthma, we factored a 0-day, 1-day, 2-day lag into the analysis. Once we completed calculations for the air pollutants' parameters by GAM, we calculated the impact of the pollutants on health. The health impact of each air pollutant was reported as a rate of increase in outpatient and emergency visits corresponding to a 10% increase in local air pollution levels. The rate of increase, rather than the number of patient visits, was considered, because downtown Taipei normally has a higher number of patient visits due to a higher number of medical facilities available, compared to other districts in the metropolitan area. The percentage change was expressed by  where  (i = 1,...,12) was a smoothing function from GAM by each district used to fit the curve; i was the district identification and further calculations required the fixed parameter to estimate the effect of the pollutants; and  was the corresponding average pollution level estimated by the Kriging method. All GAM parameters were estimated by SAS software (SAS Institute, Cary, NC). After getting each district's , we obtained the average effect and 95% confidence interval of  for the whole of Taipei City. The overall mean effect in Taipei City affected by air pollutants was constructed by the formula  where  was the average concentration of the pollutants in Taipei City. The 95% confidence interval for the percentage change was constructed by replacing  with  where  was the standard error of .

## Competing interests

The authors declare that they have no competing interests.

## Authors' contributions

TCC carried out the analysis and drafted the manuscript. JHC conceived of the study, participated in its coordination and execution. MLC participated in designing the study and interpreted the results. IFL participated in GAM analysis. CHL helped to analyze health insurance data. PHC participated in GIS analysis. WDW participated in automatic data processing. All authors read and approved the final manuscript.

## Supplementary Material

Additional file 1 **Model parameters used in the Python script**. Theses parameters used for automatic estimation of daily average concentration by Kriging method. Click here for file

Additional file 2 **Cross-validation of Kriging prediction**. Cross-validation of Kriging prediction was measured by average prediction error (PE) and root mean square standardized (RMSS). Click here for file

Additional file 3 **an illustration of 10% increase of PM_10_**. The 10% increase of air pollutant, PM_10 _was shown as an example to express the elevation of concentration in our effect's calculation. Click here for file

Additional file 4 **GAM model**. The full models of GAM were listed. Model 1 was a single pollutant model. Model 2 was a four pollutants model. Click here for file
