# Supplementary material for: Spatiotemporal analysis of air pollution and asthma patient visits in Taipei, Taiwan
Source: Int J Health Geogr. 2009 May 7;8:26. doi: 10.1186/1476-072X-8-26 (PMC2694149; doi:10.1186/1476-072X-8-26)
Supplement: Additional file 1 — Model parameters used in the Python script. Theses parameters used for automatic estimation of daily average concentration by Kriging method. [file 1476-072X-8-26-S1.pdf]

Additional files

Additional file 1 – Model parameters used in the Python script

Parameters / Air pollutants	Range	Partial Sill	Nugget	Semivariogram
SO_2	14,595	36.628	0	Gaussian
O_3	16,774	1191.1	0	Spherical
PM_{10}	7,381	233.62	0	Gaussian
NO_2	15,434	108.58	0	Gaussian
