# Supplementary material for: Spatiotemporal analysis of air pollution and asthma patient visits in Taipei, Taiwan
Source: Int J Health Geogr. 2009 May 7;8:26. doi: 10.1186/1476-072X-8-26 (PMC2694149; doi:10.1186/1476-072X-8-26)
Supplement: Additional file 2 — Cross-validation of Kriging prediction. Cross-validation of Kriging prediction was measured by average prediction error (PE) and root mean square standardized (RMSS). [file 1476-072X-8-26-S2.pdf]

Additional file 2 – Cross-validation of Kriging prediction

	PM10	SO2	O3	NO2
Average Prediction Error (PE)	2.70	0.23	-0.69	2.39
Root Mean Square Standardized (RMSS)	1.25	0.97	0.24	2.29

*Validation period: 2000/1/1-2000/1/30 (30 days)
