# Supplementary figures and images for: Spatiotemporal analysis of air pollution and asthma patient visits in Taipei, Taiwan
Source: Int J Health Geogr. 2009 May 7;8:26. doi: 10.1186/1476-072X-8-26 (PMC2694149; doi:10.1186/1476-072X-8-26)

**Additional file 3 – an illustration of 10% increase of PM<sub>10</sub>**

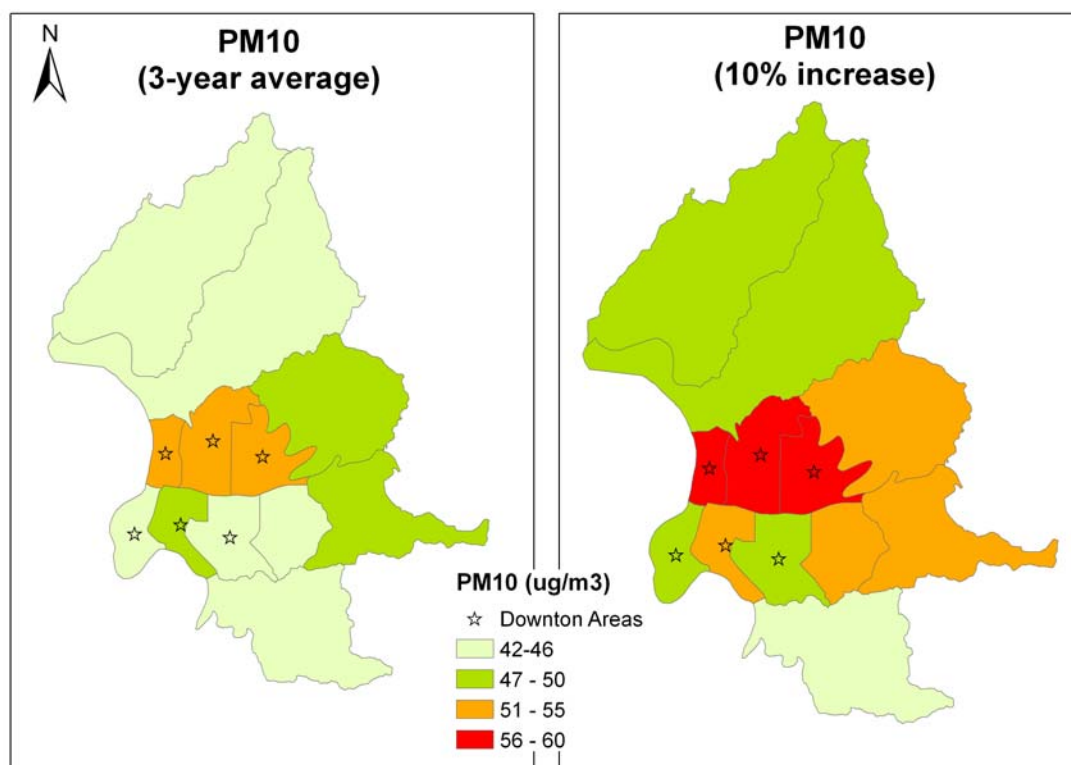

Supplement: Additional file 3 — an illustration of 10% increase of PM10. The 10% increase of air pollutant, PM10 was shown as an example to express the elevation of concentration in our effect's calculation. [file 1476-072X-8-26-S3.pdf]
