# Supplementary material for: Spatiotemporal analysis of air pollution and asthma patient visits in Taipei, Taiwan
Source: Int J Health Geogr. 2009 May 7;8:26. doi: 10.1186/1476-072X-8-26 (PMC2694149; doi:10.1186/1476-072X-8-26)
Supplement: Additional file 4 — GAM model. The full models of GAM were listed. Model 1 was a single pollutant model. Model 2 was a four pollutants model. [file 1476-072X-8-26-S4.pdf]

Additional file 4 – GAM model

$Y_{itd} \sim \text{Poisson}(\mu_{itd})$

1. Single Pollutant Model (Model 1):

$$\text{Log}(\mu_{itd})=\beta_0+\beta 1X_{weekend}+\beta 2X_{holiday}+\beta 3X_{population}+S1(X_{temperature})+S2(X_{dewpoint})+$$

$$S3(X_{weekday})+S4(X_{timeseries})+ S5(X_{onepollutant})$$

2. Four pollutants Model (Model 2):

$$\text{Log}(\mu_{itd})=\beta_0+\beta 1X_{weekend}+\beta 2X_{holiday}+\beta 3X_{population}+S1(X_{temperature})+S2(X_{dewpoint})+$$

$$S3(X_{weekday})+S4(X_{timeseries})+S5(X_{PM10})+S6(X_{SO2})+S7(X_{O3})$$

$$+S8(X_{NO2})$$

Where i=1,2,...,12 (12districts), t=2000,2001,2002(year), d=1,2,…,1096(day).

Y_{itd} : Daily asthma outpatient or emergency visits in each district.

μ_{itd} : Daily average asthma outpatient or emergency visits in each district.

$X_{weekend}$: weekend/Non-Weekend

$X_{holiday}$: Chinese New Year (5 days)

$X_{population}$: Population in four age groups in each district

$X_{temperature}$:Daily average temperature in Taipei City

$X_{dewpoint}$: Daily average dewpoint in Taipei City

$X_{weekday}$: Weekly cycle, from Monday to Sunday (1,2,…,7)

$X_{timeseries}$: Daily cycle, from first day to the last day in the study period.(1,2,…,1096)

$X_{onepollutant}$: Only one estimated daily average concentration of pollutant in each district

$X_{PM10,}$ X_{SO2}, X_{O3}, X_{NO2} : Four estimated daily average concentration of pollutants in each district

S1-S8: Smoothing function (Cubic smoothing Spline) will get approximate linear coefficient by SAS.
